# May-Thurner Syndrome Causing Unilateral Varicocele Treated With Endovascular Embolization

**DOI:** 10.7759/cureus.73490

**Published:** 2024-11-11

**Authors:** Abdulaziz Algharras, Ahmad Rchdeih, Abdulrahman m Altwigry, Mohamad Alsamal

**Affiliations:** 1 Radiology, Qassim University, Buraidah, SAU; 2 Medicine and Surgery, Sulaiman Al-Rajhi University, Al Bukayriyah, SAU; 3 Radiology, King Fahad Specialist Hospital, Buraidah, SAU; 4 Internal Medicine, Dr. Sulaiman Al Habib Medical Group, Buraidah, SAU

**Keywords:** deep venous thrombosis (dvt), endovascular embolisation, may-thurner's syndrome, varicocele treatment, vulvar varicosities

## Abstract

May-Thurner syndrome is a rare disorder characterized by the right common iliac artery overlies and compresses the left common iliac vein against the lumbar spine with or without iliofemoral deep venous thrombosis (DVT). The majority of cases are female and have been associated with the development of vulvar varicosities, particularly during pregnancy. Interestingly and very rarely, this condition has also been identified as a potential cause of varicoceles in males. This is a unique case of a 22-year-old man who had a varicocele secondary to May-Thurner syndrome, successfully treated through endovascular embolization.

## Introduction

Varicocele is a relatively common disorder in men, with a prevalence of 15% in the general population [1]. It appears predominantly on the left side.

Patients with May-Thurner syndrome end up developing iliofemoral deep venous thrombosis (DVT) because of an anatomical difference where the right common iliac artery overlies and compresses the left common iliac vein against the lumbar spine [2]. The syndrome is rarely described in males. Vulvar varicosities are the most common manifestation in women and have been linked to iliac vein blockage via the external pudendal vein, particularly during pregnancy [3]. For the same reason, it now appears to be a potential cause of varicoceles in males [4].

Males with varicocele have two main treatment options: surgical ligation and percutaneous embolization of the internal spermatic vein. Surgical ligation (varicocelectomy) has been the gold standard treatment for varicocele. It can be performed through different techniques, such as the open inguinal, subinguinal, or laparoscopic approach. On the other hand, percutaneous embolization is less invasive and is performed by an interventional radiologist. These treatment options are both effective in improving fertility and relieving symptoms [5]. Coils are the most commonly used embolic substance, with glue becoming increasingly recognized as an alternative [6]. This is a rare case of a 22-year-old man who had varicocele secondary to May-Thurner syndrome and was treated by endovascular embolization.

## Case presentation

A 22-year-old man with testicular pain was admitted to the outpatient department, presenting with an enlarged scrotum, no skin discoloration, ulcers, peripheral edema, pigmentation, or varicose veins.

**Figure 1:**
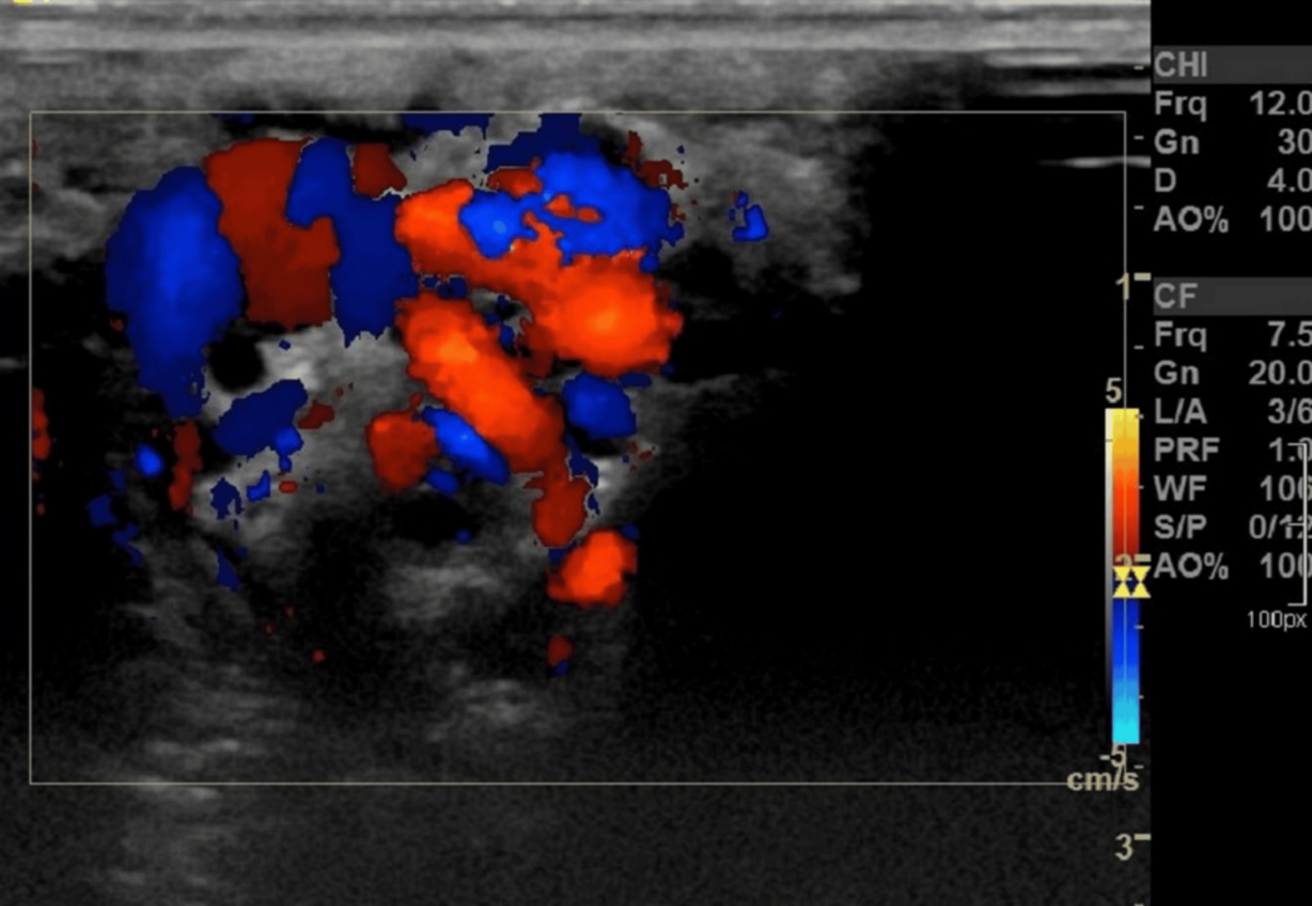
Serpiginous dilatation of pampiniform plexus veins of the left testis with reverse flow on the Valsalva maneuver.

The scrotal ultrasound revealed normal bilateral testicular size, vascularity, and parenchymal characteristics. On the left side, there are dilated serpiginous spermatic veins with vein diameters ranging from two to three millimeters, indicating a left-sided varicocele (Figure 1).

The patient was referred to the interventional radiology clinic, where the recommendation for a left-sided varicocele embolization procedure was proposed, and informed consent was duly obtained. The access site was prepared and draped in an interventional radiology suite using a maximal sterile barrier technique. Ultrasound evaluated the access site, which was found patent. An 18-gauge needle was used to puncture the right internal jugular vein, followed by a 5-French access sheath; after that, an angled catheter and guidewire were inserted into the left renal and gonadal veins.

**Figure 2:**
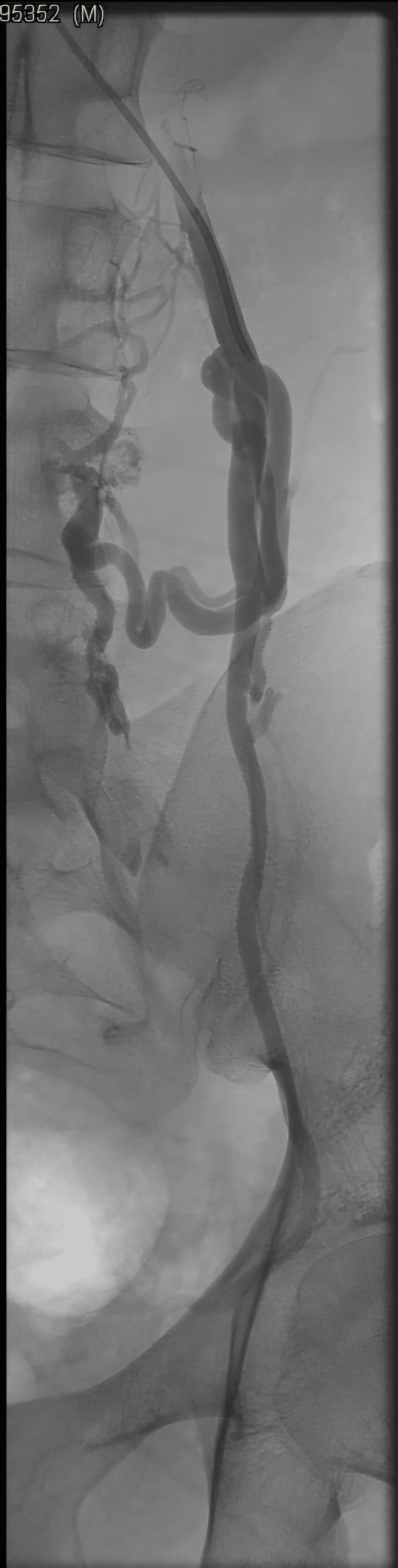
Venogram with Valsalva maneuver at the level of the left gonadal vein shows no contrast reflux below the iliolumbar collateral that is communicating with the left iliac vein.

**Figure 3:**
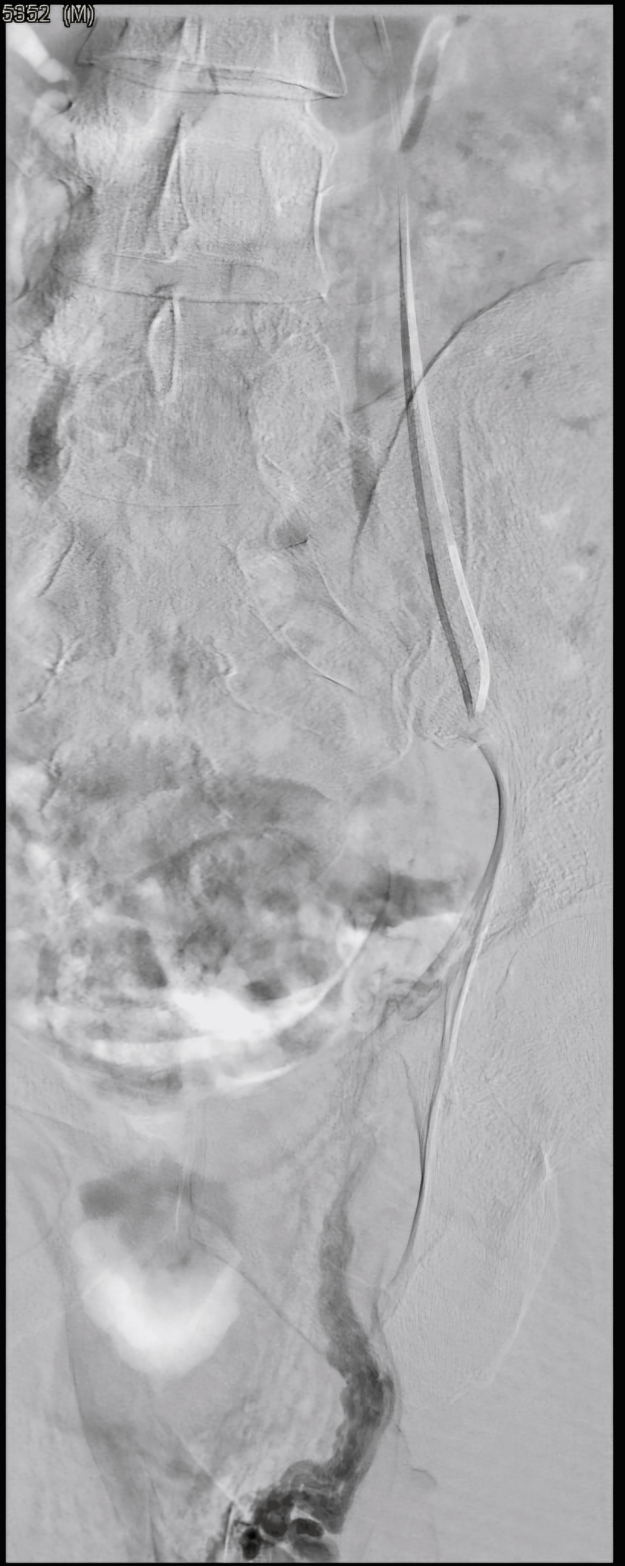
Venogram with Valsalva maneuver below the level of the iliolumbar collateral shows contrast reflux into the distal gonadal vein and pampiniform plexus.

A gonadal venogram revealed large collateral in the mid segment taking off from the iliolumbar vein in the mid-gonadal vein. A positive contrast reflux was seen in the proximal gonadal vein segment, but no reflux was seen distal to the iliolumbar vein branch (Figure 2, 3).

**Figure 4:**
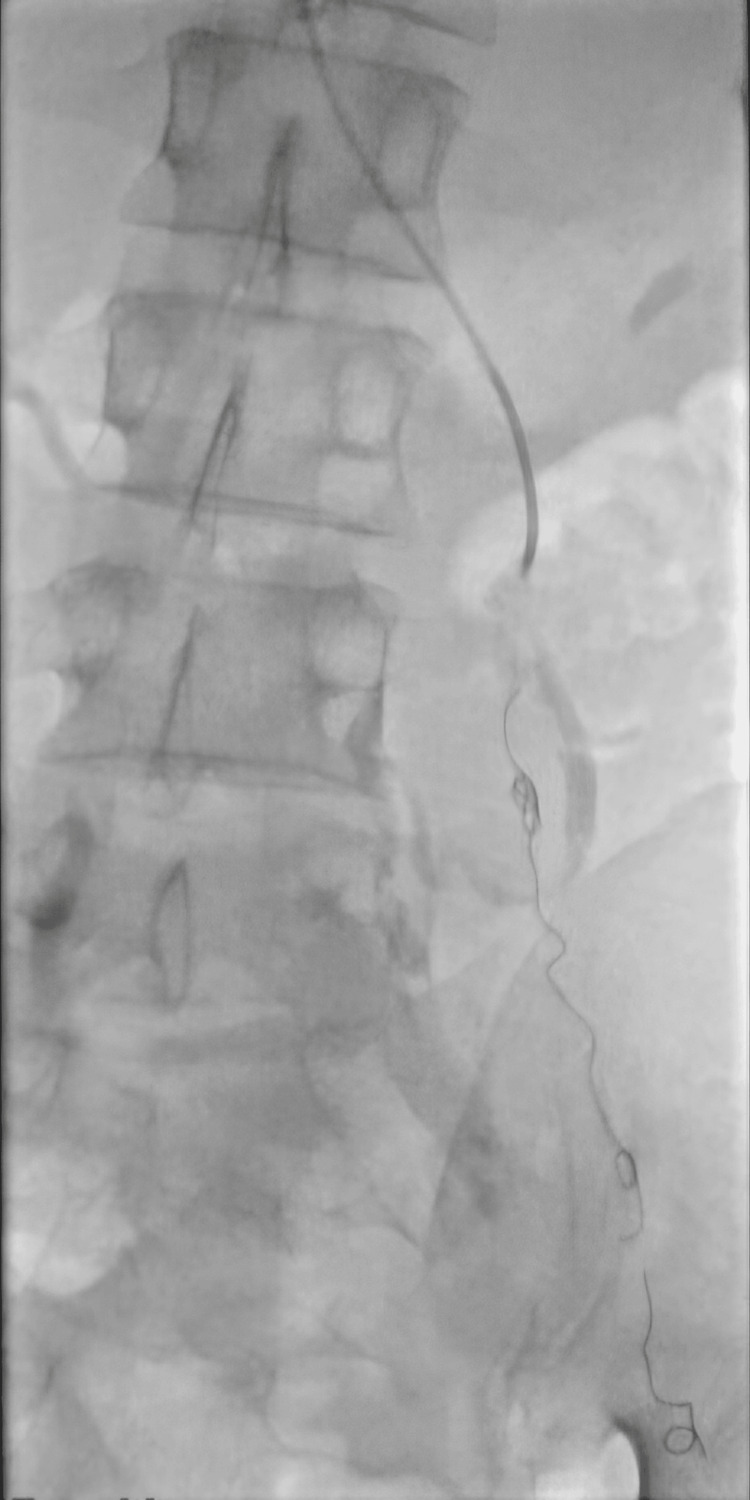
Embolization started from the pampiniform plexus to just below the iliolumbar collateral using the modified sandwich technique (STS-coils-STS-coils), with appropriate results after embolization. STS: sodium tetradecyl sulfate

The next catheter and wire were used to select the left common iliac vein. Venography confirmed severe stenosis in its origin, with multiple collaterals filling the iliolumbar collaterals, indicating May-Thurner syndrome. Overall venography findings are suggestive of May-Thurner syndrome as the cause of the varicocele. The left gonadal vein was embolized with 2 ml of sodium tetradecyl sulfate (STS) 3% foam, and 9mm and 10mm coils were deployed in a wavy pattern in the proximal gonadal vein with a few cm just below the iliolumbar collateral (Figure 4).

The procedure was well tolerated by the patient. Two hours later, the patient was discharged. Following a two-week follow-up, the patient reported that his symptoms had improved, and an ultrasound confirmed no residual reflux was identified.

## Discussion

May-Thurner syndrome, a rare complication of iliac vein compression, affects the left lower extremities and increases the risk of venous thromboembolism [7]. Notably, our patient presented with the unusual occurrence of a left-sided varicocele, an exceedingly rare complication arising from iliac vein compression. Most varicoceles are asymptomatic; if symptomatic patients experience painless scrotal edema, with a small percentage experiencing dragging pain or discomfort [1]. In our case, the patient presented with testicular dragging pain and discomfort.

Scrotal ultrasound is highly sensitive (97%) and specific (94%) for confirming clinically palpable varicoceles or looking for subclinical varicoceles [8].

On the basis of etiopathogenesis, two categories can be distinguished for varicoceles: primary and secondary [9]. Primary varicoceles are the result of venous reflux from the internal spermatic vein into the pampiniform plexus, affecting the left side (85% of cases). Secondary varicoceles are caused by an increase in testicular vein pressure, which can be caused by several factors, including hydronephrosis, hepatic cirrhosis, splenorenal shunts, abdominal and retroperitoneal neoplasms, and the nutcracker phenomenon. This case was a rare presentation of varicocele secondary to May-Thurner syndrome [10].

Two previous case reports highlighted treatment challenges in such cases. In one case, a 22-year-old man with left testicular varicocele went through multiple failed surgeries but finally found success with iliac vein stent placement following venography [11]. In another case report, a 12-year-old boy had idiopathic iliac vein blockage and an ipsilateral varicocele. He was unresponsive to surgical high ligation due to unusual varicocele formation sources and anatomical complexities that hindered the surgical treatment [3]. To the best of our knowledge, this case represents the first instance in which varicocele caused by May-Thurner syndrome was successfully treated through embolization. Varicocele embolization treatment, involving selective catheterization and blockage with sclerosant or embolic devices, is a minimally invasive, outpatient procedure with local anesthesia, seen as a viable alternative to surgery [12]. Endovascular embolization for varicocele treatment is associated with 92.4-96% success rates and recurrence rates of less than 2-4% [13]. Coil migration is a rare side effect of embolization operations [14], but our patient was recommended for continuous clinical follow-up to avoid its occurrence.

## Conclusions

Our case was a rare case of varicocele secondary to May-Thurner syndrome that was confirmed with iliac venography. Coil and sclerotherapy embolization was a good treatment choice, and our patient improved and returned to normal status.
